# Impact of COVID-19 on Affected Individuals in Iraq Focusing on Deceased Cancer Patients

**DOI:** 10.1200/GO.22.00001

**Published:** 2022-03-17

**Authors:** Nada A.S. Alwan, Faris Lami, Hanan A. Khaleel, Riyadh A. Alhilfi

**Affiliations:** ^1^ National Cancer Research Center, University of Baghdad, Baghdad, Iraq; ^2^ College of Medicine, University of Baghdad, Baghdad, Iraq; ^3^ Public Health Directorate, Ministry of Health, Baghdad, Iraq

## Abstract

Mortality because of COVID-19 infections is continuously increasing among the high-risk groups, namely, elderly patients and those with underlying comorbidities including cardiovascular diseases, diabetes, respiratory problems, and cancer. The study aimed to assess the impact of COVID-19 on affected individuals in Iraq, focusing on the characteristics of COVID-19 deceased cases, with special emphasis on cancer as the associated comorbidity.

**PATIENTS AND METHODS:**

This is a retrospective review of the data collected from 15,852 case investigation records of deceased patients with COVID-19, from all over Iraq, between March 20, 2020, and December 20, 2021. The analyzed variables included patients' age, sex, duration of stay in hospital, use of mechanical ventilation, and associated morbidities. Comparisons of having comorbidities and cancer with the characteristics were carried out using the chi-square test of independence. The chi-square test of goodness of fit was used to describe the distribution of the characteristics of the deceased COVID-19 patients; *P* values < .05 were considered statistically significant.

**RESULTS:**

Overall, 62% were ≥ 60 years with a predominance of male (63.2%). Patients with cancer were significantly younger (41.5% were ≥ 60) with no difference concerning sex distribution. Almost 70% of patients who died from COVID-19 infection had associated comorbidities. Cardiovascular diseases, diabetes, chronic obstructive pulmonary diseases, and cancer constituted 49.7%, 39.3%, 2.9%, and 1.1%, respectively. Patients with a history of cancer had a significantly longer duration of stay in hospital with no statistical association regarding the use of ventilation.

**CONCLUSION:**

In Iraq, patients with cancer infected with COVID-19 were younger and spent longer durations in the hospital before they died than patients with other comorbidities. The pandemic has revealed significant gaps in the health information and surveillance systems that demand prompt strengthening as part of the emergency preparedness.

## INTRODUCTION

After almost 2 years since the onset of the COVID-19 pandemic, countries are still enduring heterogeneous responses to the disease across various regions of the world.^1^ This global health emergency displayed significant inequality in health care access and deficit in the relevant infrastructure even among the top indexed countries on the Global Health Security Preparedness.^2^ New transmissible variants, different from those already recognized globally, are continuously emerging and circulating worldwide.^3^ As of December 21, 2021, 274,628,461 confirmed cases have been reported worldwide including 5,358,978 deaths.^4^

**CONTEXT**

**Key Objective**
What are the characteristics of COVID-19–related deaths among patients with cancer in Iraq?
**Knowledge Generated**
In about 70% of cases of COVID-19–related deaths in Iraq, the affected patients had associated comorbidities illustrated mainly as cardiovascular diseases, diabetes chronic obstructive respiratory diseases, and cancer. Patients with cancer were significantly younger and needed longer durations of stay in the hospitals before they died.
**Relevance**
To achieve an optimum description of the characteristics of COVID-19–affected cases and COVID-19–related deaths, there is an urgent need to strengthen the health information system through integration of surveillance and documentation of continuum of care as part of the emergency pandemic preparedness, specifically among patients with cancer and other comorbidities.

Mortality because of COVID-19 infections is continuously increasing among the high-risk groups, namely, elderly patients and those with underlying comorbidities comprising cardiovascular diseases (CVDs), diabetes, respiratory problems, and cancer.^5,6^ Pre-existing comorbid conditions among those patients are directly associated with hospital admissions and the need for mechanical ventilation.^7^ Concurrently, the immunosuppressed status of some patients, specifically those with a history of cancer, increases the risk of infection by the disease and subsequent complications, ultimately yielding delayed treatment, prolonged hospitalizations, and worse prognosis.^8,9^

In general, the burden of noncommunicable diseases (NCDs) including cancer continues to grow globally exerting tremendous strain on health systems specifically in developing countries that are least prepared to manage its consequences.^10^ The top four killers that account for more than 80% of all NCD deaths worldwide, in order of sequence, are CVDs, cancers, respiratory disorders, and diabetes.^11^ In Iraq, cancer is the third cause of death after heart and cerebrovascular diseases.^12^ From January 3, 2020, to December 21, 2021, the coronavirus dynamic infographic dashboard of Iraq has reported 2,090,844 cumulative cases of COVID-19 and 24,074 deaths from the disease.^13^

This study was conducted to assess the impact of COVID-19 on affected individuals in Iraq, focusing on the characteristics of the related deaths, with special emphasis on cancer as the associated comorbidity.

## PATIENTS AND METHODS

### Patients

All available data of COVID-19 deaths in Iraq were analyzed using simple descriptive statistics to identify the characteristics of COVID-19 deaths. The data set covers the period from March 20, 2020, till December 20, 2021.

### Data Source and Description

This is a retrospective review of the data set that was collected as part of the case investigation forms filled in by the respiratory unit's officers all over Iraq. The data were compiled at the Communicable Diseases Control Center/Public Health Directorate/Ministry of Health (MOH). The case investigation form has the minimum required information, such as name, age, sex, residence, date of reporting, date of death, presence of comorbidities (CVDs, diabetes, chronic obstructive pulmonary diseases [COPDs], malignancies, and others), the severity of the deceased COVID-19 patients' measures by the requirement of ventilation, delay to arrival to hospital measured by the duration of stay in hospital before death, and history of travel and contact with other patients. Data were deidentified to ensure the privacy of the patients.

### Statistical Analysis

Counts and relative frequencies were used to describe the characteristics of COVID-19 deaths in terms of age groups (< 20, 20-39, 40-59, 60-79, and 80+ years), sex (male and female), delay to arrival to hospital measured by their duration of stay in hospitals before death (died in the same day, 1-2 days, 3-9 days, 10-19 days, and 20+ days), travel history (yes, no), and history of contact with a patient with COVID-19 (yes, no). In addition, the presence of one or more comorbidities was first described as (yes, no) followed by each comorbidity (with or without the presence of others) as (yes, no). Moreover, the severity of the deceased COVID-19 patients measured by the requirement of ventilation of any kind (O_2_, continuous positive airway pressure [CPAP], and bilevel positive airway pressure) was reported as a (yes, no) variable. The chi-square test of goodness of fit was used for the univariate analysis, and *P* values lower than .05 were considered statistically significant.

In the bivariate analysis, those with comorbidities were compared with those with no comorbidities and those with malignancies were compared with those with no malignancies in terms of the aforementioned factors using the chi-square test of independence. Finally, a comparison of each of the comorbidities concerning the characteristics was done using the chi-square test of independence, too. *P* values lower than .05 were considered statistically significant.

## RESULTS

COVID-19 death database showed that there was information about 15,852 (70.0%) deceased COVID-19 patients reported from March 20, 2020, until December 20, 2021, with missing information in 1,818 (11.5%) records for the presence or lack of comorbidities. Therefore, that 11.5% were excluded during the bivariate analysis of each of the comorbidities with the characteristics of COVID-19 deaths.

Table 1 shows that the majority of the deceased COVID-19 patients were age 40-79 years (82.3%) and male (63.2%). Nearly 50% of them were age 60-79 years. About 42% of patients with COVID-19 who were admitted to the hospital died within 0-2 days. Only 34.7% were on ventilators, O_2_, or CPAP, whereas 25% had missing information regarding the use of any ventilation. Regarding the history of travel and contact with another patient with COVID-19, only 0.1% had a history of travel, whereas 10.0% had a history of contact with another patient. Another 5% of the records had missing information regarding history of travel and history of contact.

**TABLE 1:**
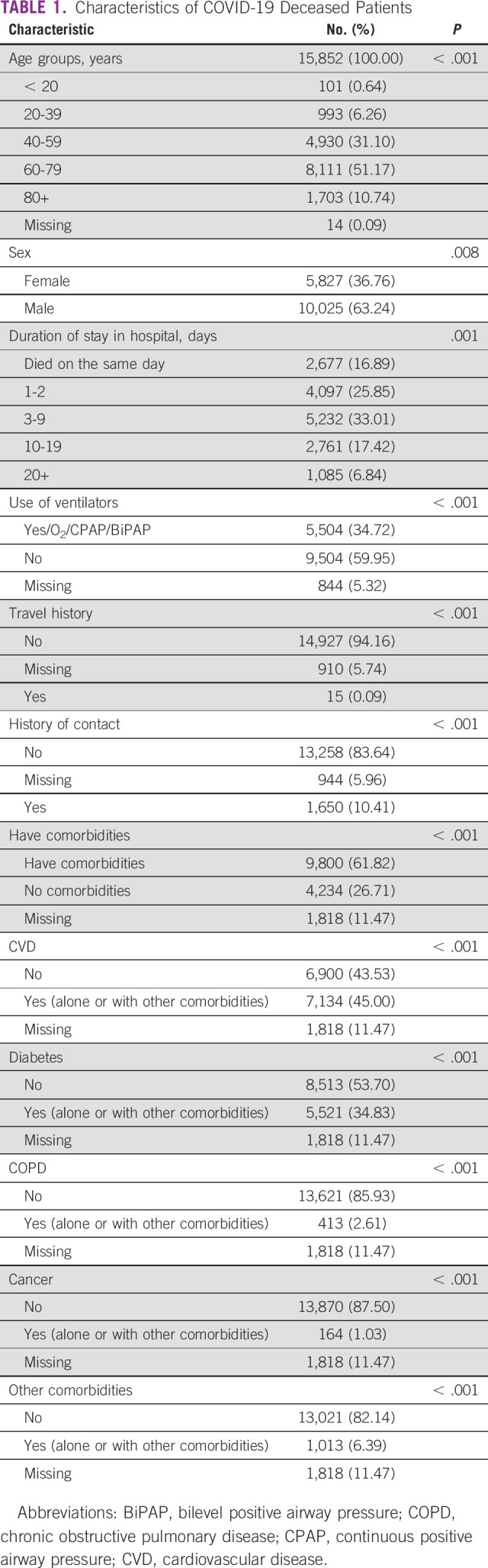
Characteristics of COVID-19 Deceased Patients

Most of the deceased COVID-19 patients had a history of one or more comorbidities (about 70% if we exclude the missing cases). That is, about 45.0% had CVDs (alone or with other comorbidities), 34.8% had diabetes (alone or with other comorbidities), 6.0% had other comorbidities, 2.6% had COPD (alone or with other comorbidities), and only 1.0% had malignancy (alone or with other comorbidities). The chi-square test of goodness of fit for characteristics of the deceased COVID-19 patients showed statistically significant *P* values for all the variables included in the study.

The comparison of the deceased patients with and without comorbidities revealed statistically significant associations between having comorbidity and age (*P* < .001), sex (*P* = .002), delayed arrival to hospital measured by the duration of stay in hospital before death (*P* = .007), and severity measured by the need to use assisted ventilation (*P* < .001; Table 2).

**TABLE 2:**
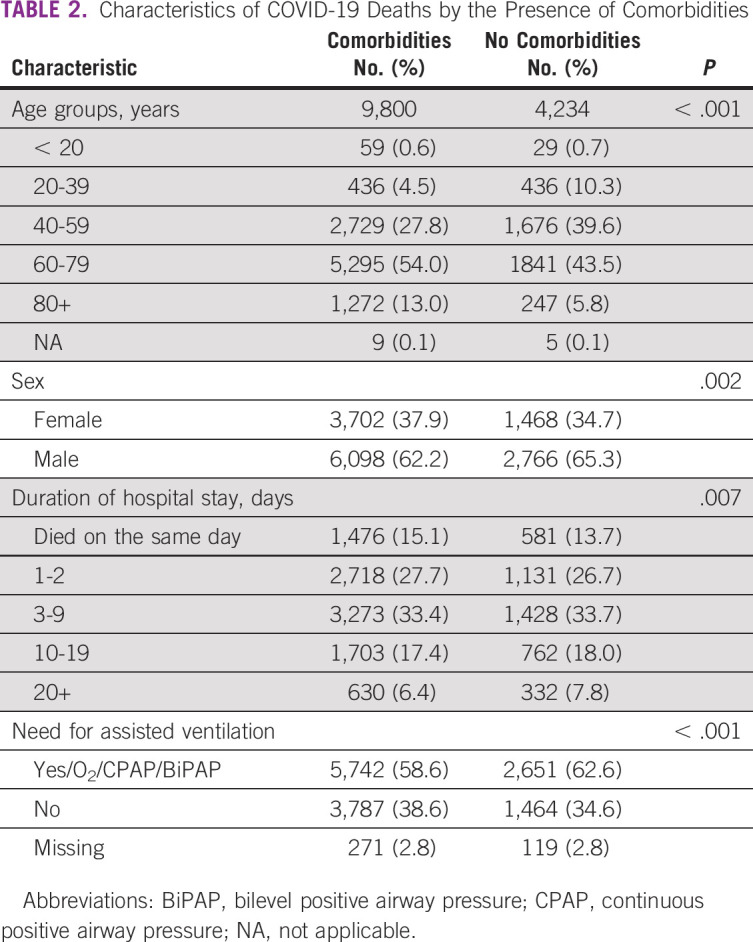
Characteristics of COVID-19 Deaths by the Presence of Comorbidities

**FIG 1:**
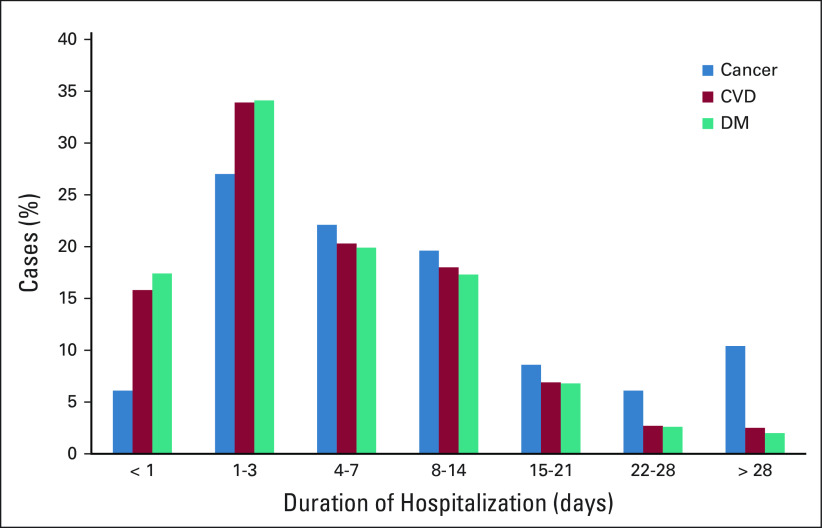
Frequency duration of hospitalization among deceased cases (cancer versus CVD and diabetes). CVD, cardiovascular disease; DV, diabetes mellitis.

Similarly, by comparing the characteristics of those with malignancy with those with no malignancy, there were statistically significant associations between having malignancy and age (*P* < .001) and the duration of stay in hospital before death (*P* < .001; Fig 1 and Table 3).

**TABLE 3:**
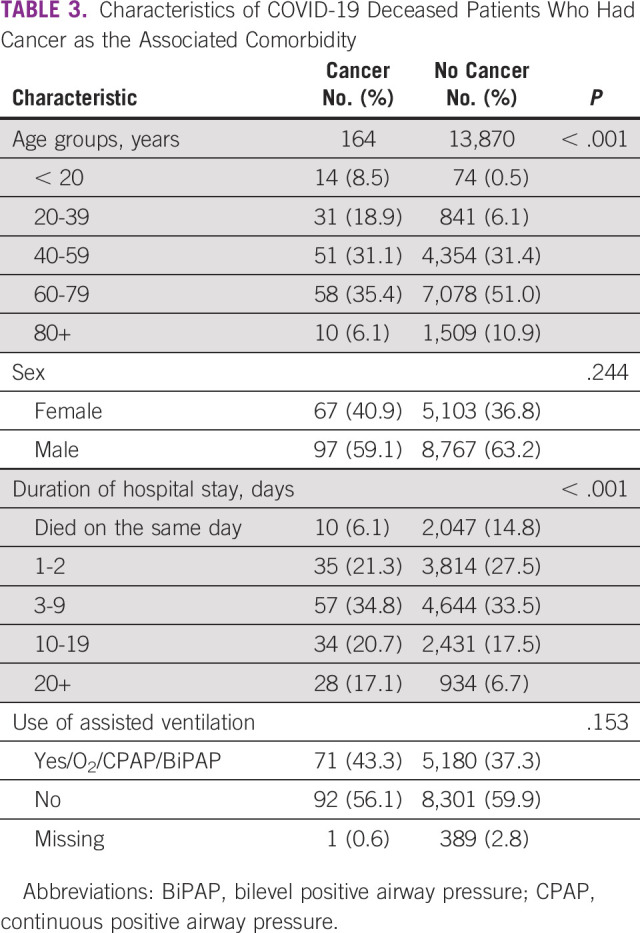
Characteristics of COVID-19 Deceased Patients Who Had Cancer as the Associated Comorbidity

Comparing each of the comorbidities with each other in terms of age, sex, duration of stay, and the use of ventilators, all showed significant associations, with the exception of patients with malignancy that revealed an insignificant relationship with sex and the need to use assisted ventilation and the insignificant association between COPD and sex (Table 4).

**TABLE 4:**
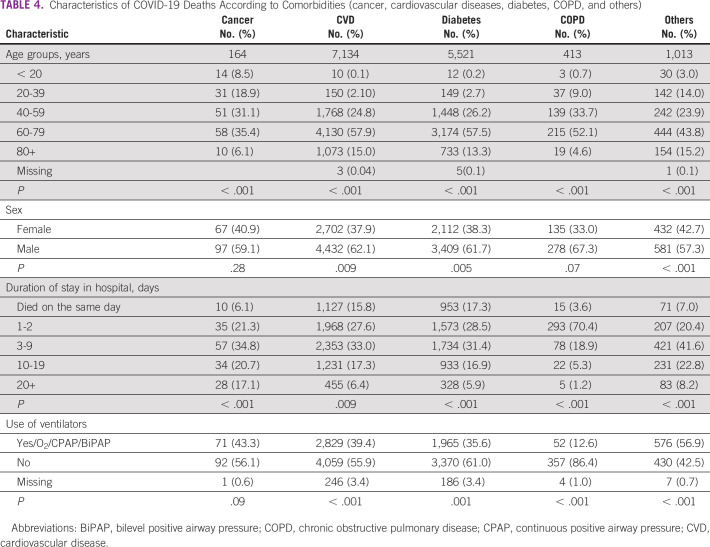
Characteristics of COVID-19 Deaths According to Comorbidities (cancer, cardiovascular diseases, diabetes, COPD, and others)

## DISCUSSION

The repeated outbreaks of COVID-19 are spreading rapidly across the globe presenting a continuous risk of emerging new mutated virulent strains that disrupt the general socioeconomic activities.^1,3^ The beginning of the pandemic was declared by the WHO in March 2020 as a public health emergency of international concern specifically in countries with vulnerable health systems.^14^ In Iraq, the long-lasting sequelae of continuous wars, sanctions, local conflicts, and displacements have yielded a fragile under-resourced health care structure that was seriously hit by the pandemic.^15,16^ The situation was exacerbated by the poor compliance of the community with the preventive measures instructed by the health authorities that exerted overwhelming pressure on the hospital bed capacity of the Iraqi MOH.^15,17^ As of December 21, 2021, the COVID-19 situation dashboard of Iraq registered an overall fatality rate of 1.1% (cumulative 24,074 deaths among 2,090,844 confirmed cases), displaying a drop in the daily number of confirmed cases as the third wave receded probably after the vaccination campaign and the rollout of the third booster dose.^13^

Our retrospective review, on the compiled data retrieved from the Communicable Diseases Control Center of the MOH, showed that approximately 70% of patients who died from COVID-19 infection in Iraq had associated comorbidities. Although this prevalence was very close to that reported in an earlier study from a neighboring Arab country,^18^ it was significantly lower than that demonstrated by other studies analyzed on COVID-19 hospitalization records in which the underlying medical conditions exceeded 90%.^19,20^ This could probably reflect the superior quality of data collected by medical chart abstraction over the standard public health surveillance in developed countries. Almost half of the comorbid conditions in this study (49.7%) were due to CVDs, whereas diabetes, COPDs, and cancer formed 39.3%, 2.9%, and 1.1%, respectively. The corresponding death rates of these NCDs among the general Iraqi population before the COVID-19 era were 27%, 4%, 2%, and 11%, respectively.^21^

A recent CDC report characterizing the comorbidities for coronavirus disease deaths among patients in Tennessee, revealed that CVDs, diabetes, cancer, and chronic lung diseases were registered in 40.6%, 36.6%, 4.3%, and 20.5%, respectively. The significantly higher rate of chronic lung diseases in their study is attributed to the fact that the term comprised asthma, emphysema, pulmonary hypertension, interstitial fibrosis, and sarcoidosis in addition to COPDs.^20^ Relatively, a multicenter retrospective cohort study on the risk factors associated with worst outcomes and deaths among Saudi patients with COVID-19 displayed that 31.2%, 20.8%, 22.1%, and 6.5% had CVDs, diabetes, chronic respiratory diseases, and cancer/immune deficiency, respectively.^18^

The overall age and gender distribution of the deceased patients in this study demonstrated that about 62% were age ≥ 60 years with a predominance of male (63.2%) versus female (36.8%). This was in line with the findings reported in earlier studies, which indicated that age and sex are well-established risk factors for COVID-19–related death.^15,18-20,22^ Similarly, in the United Kingdom, 60% of deaths from the pandemic were registered among men, but more than 90% were older than 60 years, attributed to the longer life expectancy of the British population and the competency of their national health system.^23^ On the other hand, a focus on patients with cancer in this study highlights that they were significantly younger (41.5% were age ≥ 60 years, whereas 27.4% were age < 40 years), compared with (72.9%, 2.2%), (70.9%, 2.9%), and (56.7%, 9.7%) among those who had CVDs, diabetes and COPDs, respectively. Our data did not reveal any significant difference regarding sex distribution of cancer cases contrary to that observed among cases with CVD and diabetes in this study and in contrast to the findings recorded in other surveys,^24,25^ which showed a predilection to affect men. This could probably reflect the higher incidence of cancers among female in Iraq with a male to female ratio of 1:1.3.^12^

In general, the unprecedented strain of the pandemic on the health care system has fragmented the full scope of cancer control through delaying prevention, diagnosis, and screening and by halting therapy, clinical trials, and other supportive strategies.^9^ Systematic reviews and large meta-analytical research illustrated that the weakened immune system of patients with cancer predisposes them to a higher risk of developing COVID 19 infection and eventually worse outcomes than the general population.^8,26,27^ The immune system gets compromised when cancer or its treatment procedures (chemotherapy and/or radiotherapy) affect the bone marrow impairing its capability to combat infection.^28^ The registered rate for the lethal impact of the pandemic on cancer individuals in this retrospective review (1.1%) seems relatively low when compared with other studies.^6,18,20^ This could be due to poor documentation of the full medical history, under-reporting of the cancer status by patients because of social stigma, or the reluctance to seek medical advice early after infection, leading to subsequent progression of the disease and death before arrival to the hospital. These possibilities highlight critical issues related to the effect of COVID-19 on patients with cancer in Iraq and urge the necessity to implement a robust surveillance system for tracking patients.^9,16,29^

Nevertheless, it has been interestingly reported in some studies that there was no evidence of increased mortality among patients with cancer infected by COVID-19, suggesting that the downregulated immune response in these individuals may dampen the cytokine storm and accordingly might yield better clinical outcomes.^24,30^ In an analytical cohort study on Colombian patients with cancer and COVID-19, higher mortality was only recorded in cases of active progressive or metastatic disease.^31^ Concurrently, a meta-analysis on 32 studies from Asia, Europe, and the United States concluded that elderly patients with cancer age > 65 years diagnosed with COVID-19 may not be at increased risk of death, implying that the presence of cancer may not further affect the already burdened prognosis.^26^

Compared with deceased patients with other comorbidities, patients with a history of cancer who died from the pandemic in this study spent a significantly longer duration in the hospital. It has been agreed that immunodeficient patients with cancer should be treated as an extremely vulnerable population since they are predisposed to severe symptoms of COVID-19, which require longer hospital admissions and intensive care.^7,8,24-27^ However, in contrast to other reports,^7^ our findings did not show a statistical difference among patients with cancer concerning the use of mechanical ventilation. This was consistent with the results of a National Cancer Institute–supported study, which reported that the rates of invasive mechanical ventilation are relatively low in oncology patients with COVID-19, suggesting that such individuals may not be appropriate candidates for critical care interventions.^32^ Within the same context, another observational cohort study on mechanically ventilated patients with COVID-19 demonstrated that age and a diagnosis of a solid malignant tumor were associated with 30-day mortality.^31^

In conclusion, COVID-19 introduced another threat to the fragile public health system of Iraq, which is partly dismantled through decades of wars, conflicts, and societal distrust. The pandemic has revealed significant gaps in the health information and surveillance systems that demand prompt strengthening. Our findings illustrated that older patients and those with underlying comorbid conditions were significantly associated with mortality although deceased cancer patients were younger and spent longer durations in the hospital. There is an urgent need to integrate documentation of medical history and continuum of care for patients presenting with cancer and other NCDs as part of the institutional emergency pandemic preparedness to have a clear understanding of the best practices needed to improve outcomes. Future prospective studies are warranted to explore the risk factors in COVID-19–infected individuals who present with comorbid conditions and plan for their appropriate management amid the overwhelmed health care system. Since Iraq did not introduce yet any electronic tool to identify and trace contacts, the best way to control the pandemic remains through reinforcing preventive measures, social distancing, hygiene, and vaccination.
